# NRF2 Deletion Results in Mobility Impairment in A53TSyn Model of Synucleinopathy

**DOI:** 10.3390/antiox15080926

**Published:** 2026-07-25

**Authors:** Noah Gladen-Kolarsky, Lucas Kuhnau, Wyatt Hack, Joseph F. Quinn, Nora E. Gray

**Affiliations:** 1 Department of Neurology, Oregon Health & Science University, Portland, OR 97239, USA; gladenko@ohsu.edu (N.G.-K.);; 2 Parkinson’s Disease Research Education and Clinical Care, Center (PADRECC), VA Portland Healthcare System, Portland, OR 97239, USA

**Keywords:** Parkinson’s, oxidative stress, mobility, NRF2, alpha-synuclein, mice, tyrosine hydroxylase, A53TSyn, gait, open field

## Abstract

Parkinson’s Disease (PD) is the second most diagnosed neurological disorder globally, affecting millions of people worldwide. Oxidative stress is implicated in the progression of PD, yet its direct effects on motor function, particularly in the context of synucleinopathy, are not fully understood. Here, we investigated the effects of the loss of the antioxidant regulatory transcription factor NRF2 in the A53TSyn mouse model of synucleinopathy. Motor function was evaluated in separate cohorts of A53TSyn mice without NRF2 (A53TSyn/NRF2KO), as well as A53TSyn mice expressing NRF2 (A53TSyn/NRF2+) and healthy wild-type (WT) mice at four, six, and eight months of age. The overall mobility decreased in A53TSyn/NRF2KO mice relative to WT mice at all ages. Significant alterations in gait were also apparent in A53TSyn/NRF2KO mice compared to A53TSyn mice without NRF2 deletion. Expression of tyrosine hydroxylase (TH) was also quantified in the brains of those mice. While there were no differences in cortical pSyn expression between A53TSyn/NRF2+ and A53TSyn/NRF2KO mice, a reduction in TH abundance in the striatum was evident in A53TSyn/NRF2KO mice at all ages. In summary, our data suggest that NRF2 plays a role in maintaining mobility and gait in the context of synucleinopathy and may represent a therapeutic target to mitigate mobility decline in PD-affected individuals.

## 1. Introduction

Parkinson’s Disease (PD) is a progressive neurological disorder which typically manifests clinically through motor symptoms including tremor, bradykinesia, and postural imbalance [1]. An estimated 8.5 to 10 million people are affected by PD worldwide, including 1 to 1.5 million in the United States with an incidence rate of nearly 90,000 individuals yearly [2]. The prevalence of PD has doubled in the past 25 years and PD is the second most common neurodegenerative disease worldwide [2]. Despite these growing numbers, therapeutic options for PD remain limited, owing in part to an incomplete understanding of the pathogenesis and progression of the disease.

Significant cell atrophy paired with loss of dopaminergic neurons is evident in the PD brain within the substantia nigra pars compacta [2]. This cell loss results in decreased dopamine levels within the striatum, which are correlated with the progressive motor deterioration observed in PD. Another pathological characteristic of PD is the presence of Lewy bodies, a major component of which is misfolded aggregates of the phosphorylated alpha-synuclein (pSyn) protein. This aggregated pSyn is thought to contribute to the neurodegeneration seen in PD, although the mechanisms by which this occurs remain to be elucidated.

One mechanism through which aggregated pSyn evokes neurotoxicity is through oxidative stress. The damage caused by pSyn to mitochondria can result in increased generation of reactive oxygen species (ROS) which can further promote the misfolding and aggregation of pSyn [3,4]. Genetic mutations associated with PD also impact mitochondrial function, leading to increased ROS generation [5]. Oxidative stress is an early event in the PD brain [6] and is widely thought to contribute to disease progression [7,8,9]. Multiple studies have demonstrated that PD patients have elevated levels of serum ROS [5,10,11]. Increased oxidative damage to membranes, DNA, and protein is seen in the post-mortem PD brain [12]. This increased oxidative damage is also recapitulated in rodent models of PD [13].

Nuclear factor erythroid 2-related factor 2 (NFE2L2, also known as NRF2) is a key transcription factor which regulates the expression of antioxidant enzymes [14]. These antioxidant enzymes play an important role in regulating cellular redox homeostasis, the disruption of which can lead to deleterious oxidative injury to biological macromolecules [15]. There is mounting evidence that NRF2 plays an important protective role in PD progression. A functional haplotype in the *NFE2L2* promoter region that results in increased transcription has been associated with decreased risk and delayed onset of the disease in humans, and evidence for a beneficial effect of NRF2 in PD has been accumulating in animal models as well [16,17,18,19]. In two toxin-induced models of PD using MPTP (1-methyl-4-phenyl-1,2,3,6-tetrahydropyridine), it was demonstrated that activation of the NRF2 pathway mitigated neuronal loss [20,21]. Conversely, NRF2 deficiency has been shown to exacerbate sensitivity to MPTP [22,23]. The protective action of NRF2 has also been demonstrated in vitro, where activation of NRF2 in glial cells has been shown to promote the survival of dopamine neurons [24] and to prevent cellular senescence in vitro [25]. However, whether NRF2 contributes to the maintenance of motor function in PD is not fully understood.

This study aimed to address this question using the A53TSyn mouse model of synucleinopathy in which NRF2 was knocked out to examine possible additive effects between the two mutations. A53TSyn mice have widespread alpha-synuclein inclusions in the brain and spinal cord, and exhibit common characteristics seen in human synucleinopathies, like PD, such as neuroinflammation and oxidative stress [26,27]. Motor deficits have been described in this model, as well as age-dependent dopamine cell loss [28]. The slower timeline of neurodegeneration relative to toxin models like MPTP allows for longitudinal analysis of changes that contribute to motor dysfunction, making it an ideal model for the present study. Here we describe the effects of loss of NRF2 in four, six, and eight month-old A53TSyn animals.

## 2. Materials and Methods

### 2.1. Animals

All experiments were performed according to the NIH Guidelines for the Care and Use of Laboratory Animals and were approved by the Institutional Animal Care and Use Committee of the Veterans Administration Portland Health Care System (VAPORHCS; IACUC #4688). A53TSyn transgenic mice (Genotype: B6; C3-Tg (Prnp-SNCA*A53T)83Vle/J; “A53TSyn/NRF2+”) and NRF2 knockout (Genotype: B6.129X1-*Nfe2l2^tm1Ywk^*/J; “NRF2KO”) mice, both on a C57BL6 background, were purchased from Jackson Laboratory (Bar Harbor, ME, USA) (Strain #004479 and Strain #017009). In order to generate mice for this study that were both homozygous for the A53TSyn mutation and homozygous for the NRF2 knockout (A53TSYN/NRF2KO), heterozygous A53TSyn mice on a C57BL6 background that expressed NRF2 were paired with mice homozygous for the NRF2 deletion also on a C57BL6 background. Hemizygous offspring were then paired to produce A53TSyn/NRF2KO mice. Complete loss of the NRF2 allele and homozygous presence of the A53TSyn mutation were confirmed by independent genotyping analysis conducted by Transnetyx (Cordova, TN, USA) by qPCR using validated primers. For the NRF2KO genotyping, samples were held at 60 °C for 1 min, then 95 °C for 10 min, followed by 40 cycles at 95 °C for 15 s and 60 °C for 1 min. The same temperature settings were used for the A53TSyn genotyping except that the 95 °C hold was for 5 min. The A53TSyn/NRF2+ mice for this experiment were generated by pairing heterozygous A53TSyn mice on a C57BL6 background. A diagram of the breeding scheme can be found in Supplementary Figure S1. C57BL6 mice (WT) were obtained from the National Institute of Aging (NIA) to be used as the WT control group. Experimental sample size was determined based on previous studies that our group has performed with A53TSyn mice [29], and no animals for which data were collected were excluded from the study. Mice were housed in a climate-controlled facility with a 12 h light/dark cycle, fed *ad libitum*, and monitored at least 2×/week for any changes in physical condition or behavior.

**Figure 1:**
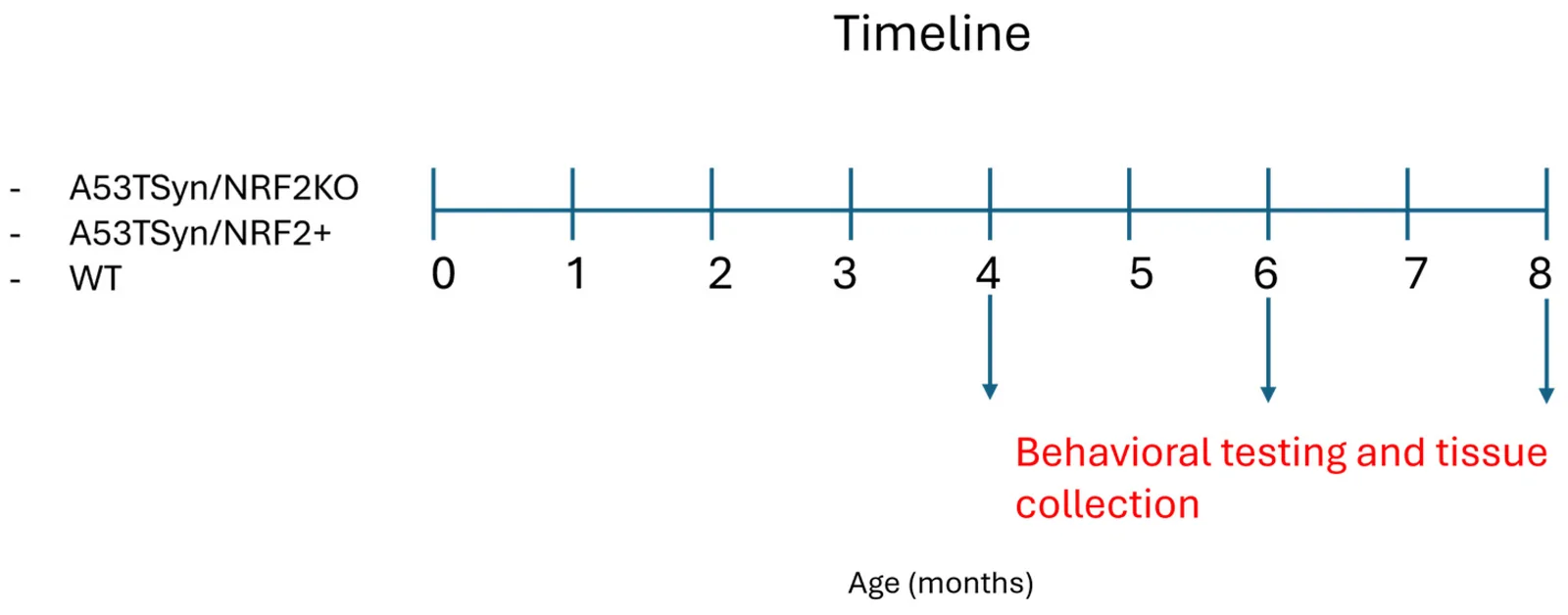
Study Design: C57BL6 (WT), A53TSyn/NRF2+, and A53TSyn/NRF2KO mice were aged to 4, 6, and 8 MO, and behavioral testing was carried out at each age point followed by tissue collection.

At 4, 6, and 8 months of age, A53TSyn/NRF2+, A53TSyn/NRF2KO, and WT animals underwent behavioral testing, which included open field and DigiGait (Figure 1). Behavioral tests were always conducted in the same order. At the conclusion of behavioral testing, mice were euthanized according to VAPORHCS guidelines, and tissue was collected for immunohistochemical analysis. The primary endpoint was performance in the open field test. All groups included approximately equal numbers of male and female mice (Supplementary Figure S2); the experimental unit was a single animal. Investigators were blinded to experimental conditions following data collection and prior to results analysis. Data were obtained from separate cohorts of mice analyzed at either 4, 6, or 8 months (MO) of age.

### 2.2. Open Field Test (OF)

The OF behavioral test was used to assess motor function and anxiety-related behavior in mice. For this test, animals were placed in a 40 × 40 × 40 inch square enclosure and allowed to explore freely for 5 min per trial. Two trials were performed on each of two consecutive days of the test, constituting a total of 20 min of open field testing, with a 15 min rest period for each animal between consecutive runs. Time spent immobile, distance traveled, and time in different locations of the arena were quantified automatically via an overhead camera in AnyMaze software (Stoelting Co., Wood Dale, IL, USA; Version 7.07) and averaged across all four 5 min trials. Increased time in the center reflects reduced anxiety, while increased time in the periphery indicates higher anxiety. The enclosure was sterilized with 70% ethanol between runs to disinfect it and remove scent markers.

### 2.3. DigiGait

DigiGait is a digital gait analysis tool (DigiGait, Mouse Specifics Inc., Framingham, MA, USA). Mice were placed in a chamber on top of a transparent treadmill and moved forward at a speed of 20 cm/s on a 15° decline while a camera (Basler A602 camera, 150 fps) captured video from below the animal. Video of a continuous run was captured for 4–8 s. Footage was thresholded to indicate initial paw placement to the software, and gait data were then generated automatically by the program. Occasionally, mice would not move on the treadmill, preventing automated gait analysis—these animals were excluded from the final analysis (*n* = 11 excluded). Post-quantification error checking was subsequently performed for each video. Metrics for gait were generated by the software from the digitized indices.

### 2.4. Immunohistochemistry

Right hemispheres were collected and immediately placed into 4% paraformaldehyde, phosphate-buffered saline (PBS), 15% and 30% sucrose solutions for 24 h each, then stored at −80 °C for sectioning. Coronal sections (40 um) were obtained by slicing right hemisphere samples held at −20 °C in Optimal Cutting Temperature (O.C.T.) compound (Sakura Finetek, Torrance, CA, USA) on a freezing microtome. Sections were stored in a sectioning solution containing 15% glycerol, 10% Tris-HCl buffered saline (TBS), diluted in diH_2_O before further processing.

During immunostaining, sections were placed in a quenching solution containing 30% methanol, 10% hydrogen peroxide, and 10% TBS for endogenous catalase activity, then subsequently blocked in a solution containing 2% bovine serum albumin, 10% horse serum, 2% Triton X, 10% 10× TBS, diluted in diH_2_O. Sections were then incubated with one of the following antibodies diluted in the blocking solution previously described: anti-tyrosine hydroxylase polyclonal (Thermo Fisher, Waltham, MA, USA; CAT #PA5-85167) and anti-alpha-synuclein, monoclonal, phospho-S129 (Abcam, Waltham, MA, USA; CAT #AB51253). Target binding was visualized using biotinylated secondary antibodies, the VECTASTAIN ABC-HRP system (Vector Laboratories, Inc., Newark, CA, USA), and DAB chromogen (Sigma-Aldrich, St. Louis, MO, USA; CAT #D4418).

ImageJ software (National Institutes of Health, Bethesda, MD, USA; Version 1.54s) was used to quantify antibody staining. Images were converted to greyscale, and a tracing tool was used to outline the region of interest. Contrast thresholding was adjusted according to background staining, and stains were quantified from three sections at different depths from each right hemisphere sample. The extent of staining was expressed as a percentage of the area stained versus the total area of the region of interest, and mean values for each sample were calculated from the three sections analyzed.

### 2.5. Statistical Analysis

All bar graphs show error bars representing the standard error of the mean (SEM). Statistical analyses were performed using a two-way ANOVA to assess the effects of genotype and age. If there was no significant effect of one variable, then data across that variable were combined for each genotype, and a one-way ANOVA followed by Tukey’s multiple comparisons test was conducted. A linear regression analysis was performed on Overlap Distance slopes to determine whether the slopes were significantly non-zero. All statistical analyses were performed in GraphPad Prism 10 software (GraphPad Software, Inc., Boston, MA, USA).

## 3. Results

### 3.1. Loss of NRF2 Exacerbates Tyrosine Hydroxylase (TH) Expression in A53TSyn Mice

**Figure 2:**
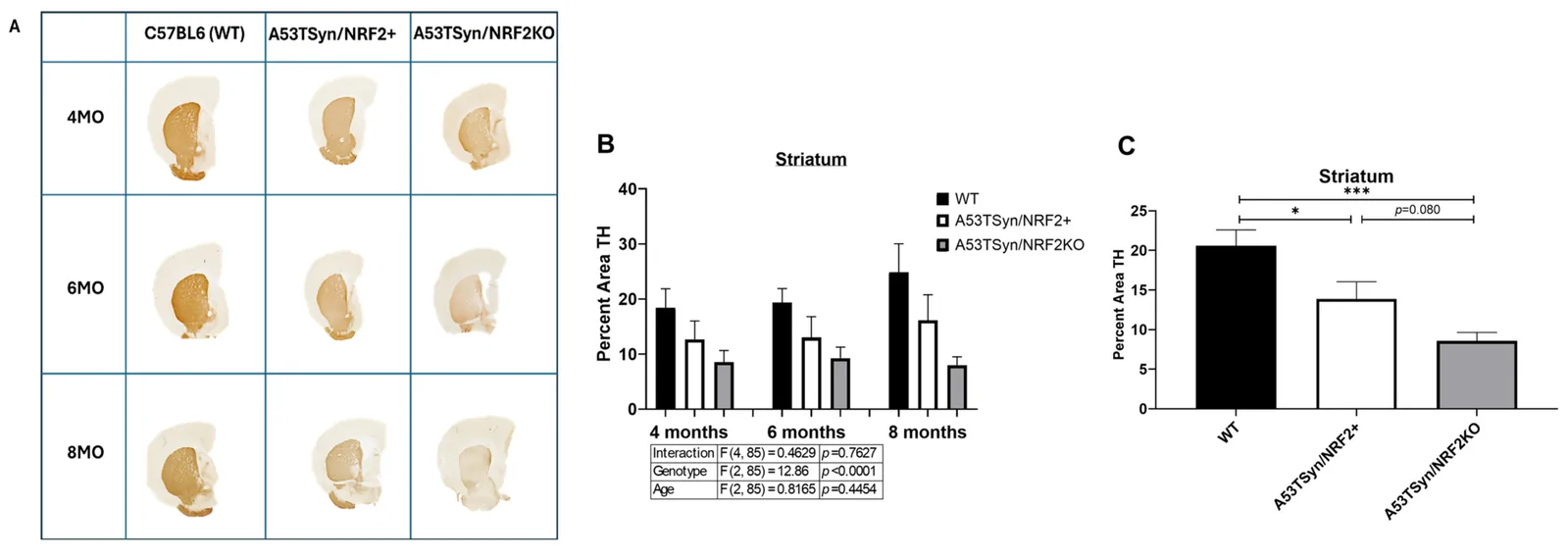
Striatal TH Expression is Reduced in A53TSyn/NRF2KO Mice. (**A**) Representative images of tyrosine hydroxylase immunohistochemical staining at 4, 6 and 8 months. (**B**) Striatal TH expression by age and genotype. Expression was significantly altered by genotype but not by age. (**C**) Striatal TH expression by genotype (ages combined) was reduced in both A53TSyn/NRF2+ and A53TSyn/NRF2KO animals compared to WT. Error bars indicate SEM. * *p* < 0.05, “***”*p* < 0.001 (ANOVA), 4MO WT *n* = 8; 4MO A53TSyn/NRF2+ *n* = 9; 4MO A53TSyn/NRF2KO *n* = 13; 6MO WT *n* = 14; 6MO A53TSyn/NRF2+ *n* = 9; 6MO A53TSyn/NRF2KO *n* = 11; 8MO WT *n* = 8; 8MO A53TSyn/NRF2+ *n* = 7; 8MO A53TSyn/NRF2KO *n* = 12.

The expression of TH was quantified in the striatum of mice at four, six, and eight months of age (Figure 2A). Analysis by two-way ANOVA found a significant effect of genotype but not of age, nor was there a significant interaction between age and genotype (Figure 2B). Analysis by genotype with all ages combined found a significant reduction in TH expression between WT and both A53TSyn/NRF2+ and A53TSyn/NRF2KO mice (Figure 2C) and a trend towards even further reduced expression in A53TSyn/NRF2KO mice compared to A53TSyn/NRF2+ (*p* = 0.080). A similar pattern of TH expression was observed in the substantia nigra (Supplementary Figure S3).

### 3.2. NRF2 Deletion Does Not Affect Cortical Phosphorylated Alpha-Synuclein (pSyn) Aggregation

**Figure 3:**
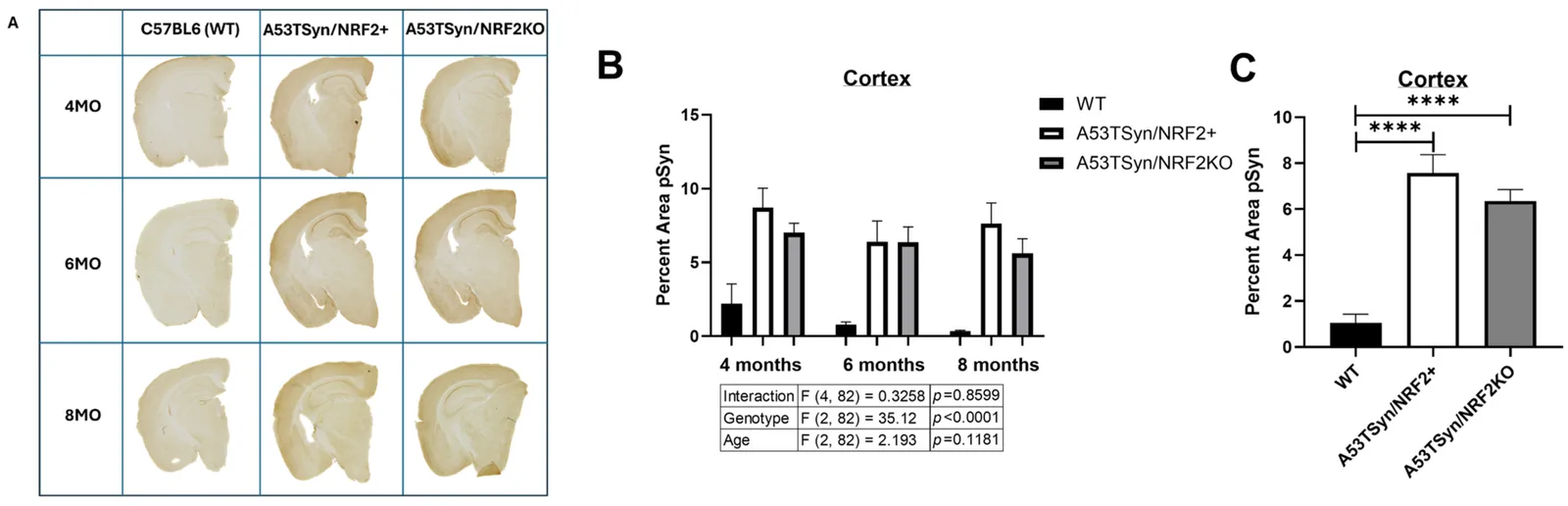
Cortical pSyn Aggregation is Unaffected in A53TSyn-NRF2KO Mice. (**A**) Representative images of phosphorylated alpha-synuclein immunohistochemical staining at 4, 6 and 8 months. (**B**) Cortical pSyn expression by age and genotype. The genotype effect was significant but not age nor the interaction. (**C**) Cortical pSyn expression by genotype, ages combined. Error bars are SEM. **** *p* < 0.0001 (ANOVA); 4MO WT *n* = 8; 4MO A53TSyn/NRF2+ *n* = 9; 4MO A53TSyn/NRF2KO *n* = 13; 6MO WT *n* = 14; 6MO A53TSyn/NRF2+ *n* = 9; 6MO A53TSyn/NRF2KO *n* = 11; 8MO WT *n* = 8; 8MO A53TSyn/NRF2+ *n* = 7; 8MO A53TSyn/NRF2KO *n* = 12.

The abundance of pSyn was assessed in the cortex of mice at four, six, and eight months of age (Figure 3A). There was a significant effect of genotype but not of age, and the interaction between age and genotype was also nonsignificant (Figure 3B). No differences in cortical pSyn expression were found between A53TSyn/NRF2+ and A53Tsyn/NRF2KO when all ages were combined (Figure 3C), but both were significantly different from WT (*p* < 0.0001 for both comparisons). There were similarly no additive effects of the loss of NRF2 and the A53TSyn overexpression on pSyn expression in either the hippocampus or the striatum of A53TSyn/NRF2KO mice compared to A53TSyn/NRF2+ mice (Supplementary Figure S4).

### 3.3. A53TSyn/NRF2KO Mice Show Impaired Overall Mobility and Possible Effects on Anxiety-Related Behavior

**Figure 4:**
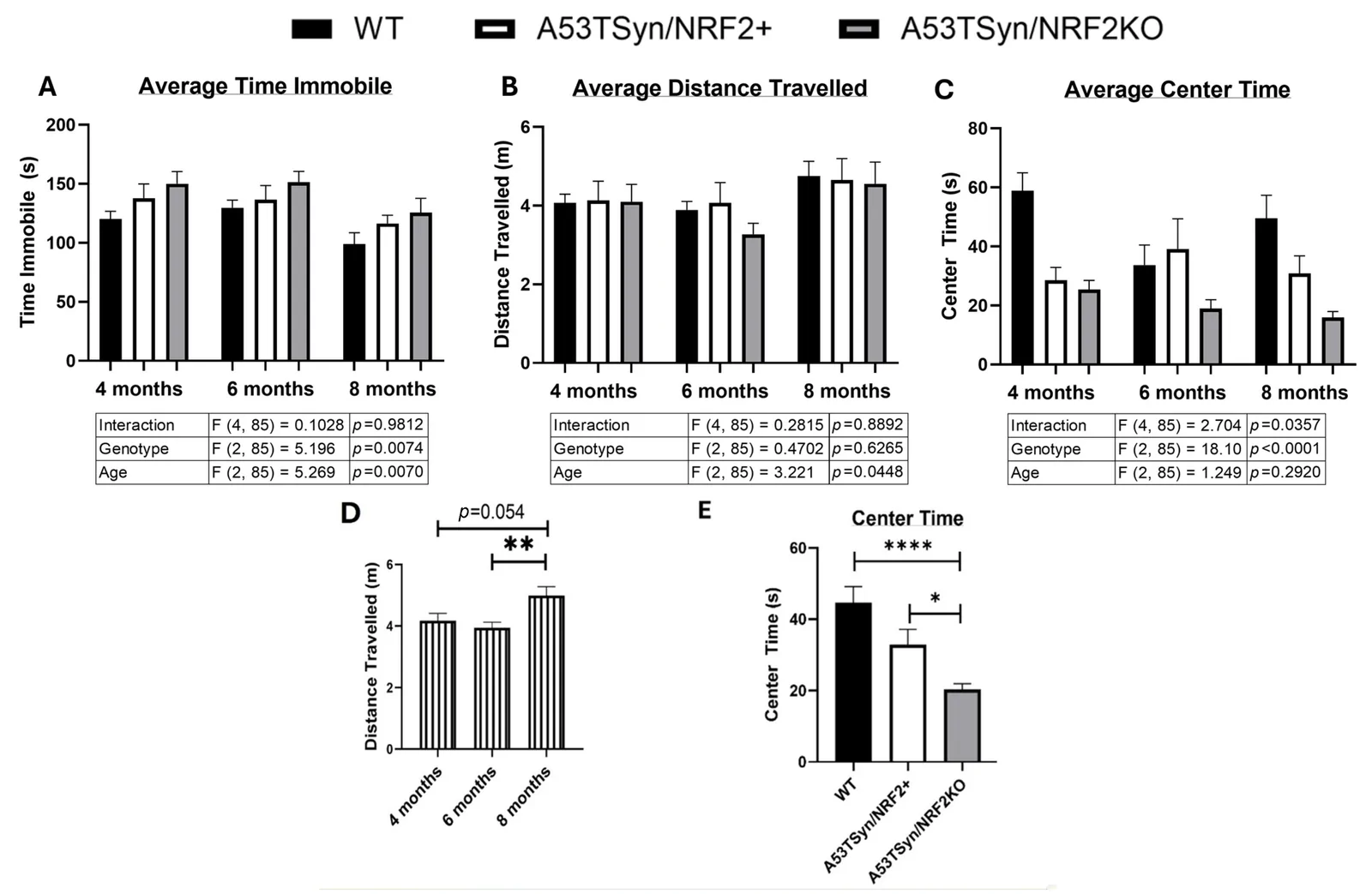
Loss of NRF2 Exacerbates Behavioral Abnormalities in Open Field (OF) Test. (**A**) Average time immobile in seconds for each age and genotype. (**B**) Average distance traveled for each age and genotype. (**C**) Average time spent in center in seconds for each age and genotype. (**D**) Average distance in meters traveled by age (genotypes combined). (**E**) Average center time in seconds by genotypes (ages combined). Error bars reflect SEM. * *p* < 0.05, ** *p* < 0.01, **** *p* < 0.0001 (ANOVA); 4MO WT *n* = 8; 4MO A53TSyn/NRF2+ *n* = 9; 4MO A53TSyn/NRF2KO *n* = 13; 6MO WT *n* = 14; 6MO A53TSyn/NRF2+ *n* = 9; 6MO A53TSyn/NRF2KO *n* = 11; 8MO WT *n* = 8; 8MO A53TSyn/NRF2+ *n* = 7; 8MO A53TSyn/NRF2KO *n* = 12.

There was a significant effect of both age (*p* = 0.007) and genotype (*p* = 0.007) for average time immobile on the open field test (Figure 4A), although the interaction was not significant. There was a significant effect of age (*p* = 0.006) for total distance traveled, but no significant effect of genotype or the interaction (Figure 4B). Aggregating all genotypes together by age (Figure 4D) revealed that the eight-month group traveled significantly more than the six-month group (*p* = 0.005) and the four-month group (*p* = 0.0102).

Time in the center during the open field test was also recorded. Reduced center time is associated with increased anxiety-related behavior. There was a significant effect of genotype (*p* < 0.0001) but not of age, although the interaction between age and genotype was found to be significant (*p* = 0.036; Figure 4C). A53TSyn/NRF2KO mice spent significantly less time in the center relative to both WT (*p* < 0.0001) and A53TSyn/NRF2+ (*p* = 0.028) when all ages were combined (Figure 4E).

### 3.4. Left Ipsilateral Fore/Hind Limb Overlap Progressively Declines with Age in A53TSyn/NRF2KO Mice

DigiGait was used to assess gait. This system quantifies 43 individual spatial and temporal gait parameters in each paw via ventral plane videography. Changes in metrics were apparent between genotypes at each age point but were not always consistent across all four paws (Supplementary Figure S5). In these trials, 11 mice of 94 were excluded from the analysis because they would not move on the treadmill, but these excluded animals were evenly distributed between the different experimental groups.

Overlap Distance (OD) measures the horizontal distance between the fore and hind paws ipsilaterally during stepping in centimeters. Because OD is measured by the distance between the centroids of these two paws; if the paws do not overlap, the values are negative. A decrease in overlap distance is commonly associated with motor deterioration, as it indicates that the hind paw is not advancing forward to meet or overlap with the placement of the fore paw as is normal in healthy mice.

**Figure 5:**
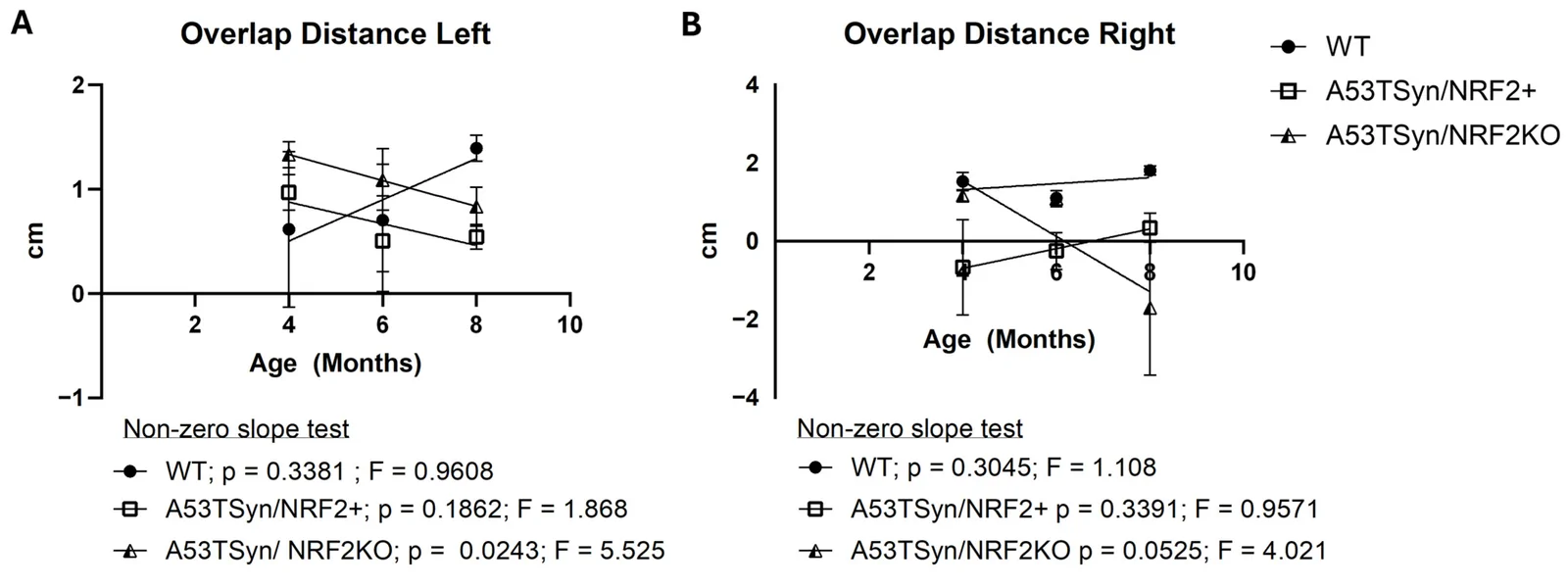
Left Overlap Distance (OD) Progressively Declines with Age. Change in OD over time was determined through linear regression analysis. (**A**) On the left side, only A53TSyn/NRF2KO showed a significant non-zero negative slope (*p* = 0.024). (**B**) On the right side, no significant changes in OD were observed through non-zero linear regression analysis, although the NRF2KO group approached significance (*p* = 0.053); Error bars reflect SEM. 4MO WT *n* = 7; 4MO A53TSyn/NRF2+ *n* = 7; 4MO A53TSyn/NRF2KO *n* = 14; 6MO WT *n* = 8; 6MO A53TSyn/NRF2+ *n* = 8; 6MO A53TSyn/NRF2KO *n* = 11; 8MO WT *n* = 8; 8MO A53TSyn/NRF2+ *n* = 8; 8MO A53TSyn/NRF2KO *n* = 13.

A reduction in OD was observed in A53TSyn/NFR2KO mice over time on both sides in an age-dependent manner, although the slope of this change over time was only statistically different from 0 on the left side (*p* = 0.024), while on the right side, the *p* value of the slope was 0.053. There was a modest trend towards a decline in OD also evident in A53TSyn/NRF2+ mice with age on the left side (*p* = 0.1862; Figure 5A) but not on the right side.

### 3.5. Diminished Gait Fluidity Is Evident in A53TSyn/NRF2KO Mice at All Ages

Changes in the minimum and maximum rate of change in paw contact area (A) on the DigiGait treadmill over time (T; dA/dT) during the propulsion and braking phases of the step motion can reflect gait fluidity. The minimum change in area of contact with the treadmill over time occurs during the “propulsion” phase of the step motion, wherein the animal is pushing the paw off the belt, and paw area of contact decreases over time. Conversely, the maximum change in area of contact with the belt occurs as the paw makes initial contact in the “braking” phase of the step motion, as the paw area of contact increases over time.

**Figure 6:**
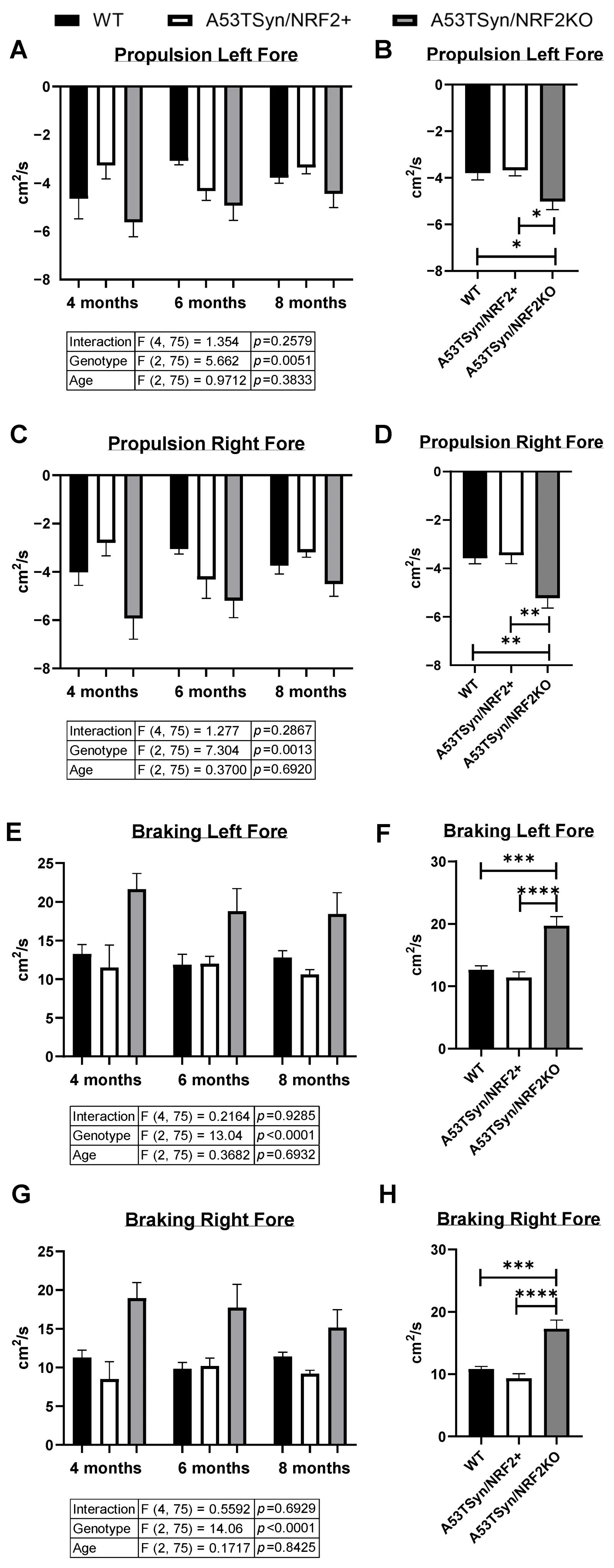
Fore Paw Gait Fluidity is Altered during Brake and Propel Step Movement. (**A**) Change in paw area contact over time (dA/dT) values during propulsion for the left fore paw by age and genotype. Genotype, but not age or the interaction, was found to be the only significant effect. (**B**) Aggregated propulsion values for the left fore paw from all ages. (**C**) Propulsion values for the right fore paw by age and genotype. Genotype was the only comparison for which there was a significant effect detected. (**D**) Aggregated propulsion values for the right fore paw from all ages. (**E**) Braking values for the left fore paw by age and genotype. Although genotype was found to be significantly different, neither age nor the interaction were found to be significant. (**F**) Aggregated braking values for the left fore paw from all ages. (**G**) Braking values for the right fore paw by age and genotype. Age and interaction were nonsignificant, while the genotype effect was found to be significant. (**H**) Aggregated braking values for the right fore paw from all ages. Error bars reflect SEM. * *p* < 0.05, ** *p* < 0.01, *** *p* < 0.001, **** *p* < 0.0001 (ANOVA) 4MO WT *n* = 7; 4MO A53TSyn/NRF2+ *n* = 7; 4MO A53TSyn/NRF2KO *n* = 14; 6MO WT *n* = 8; 6MO A53TSyn/NRF2+ *n* = 8; 6MO A53TSyn/NRF2KO *n* = 11; 8MO WT *n* = 8; 8MO A53TSyn/NRF2+ *n* = 8; 8MO A53TSyn/NRF2KO *n* = 13.

Although no differences were detected in propulsion or braking in the hind paws at any age (Supplementary Figure S6), significant alterations in these gait metrics were apparent in the fore paws of A53TSyn/NRF2KO mice (Figure 6A–H). There was a significant genotype effect observed in minimum dA/dT in the propulsion phase for both the left (*p* = 0.005) and right (*p* = 0.001) fore paws, but not of age, and the interaction was also not significant (Figure 6A,C). During the braking phase in both the left and right forepaws (Figure 6E,G), the results were similar, with a significant genotype effect (*p* < 0.0001 for both right and left), but not a significant effect of age nor an interaction. In both fore paws, the A53TSyn/NRF2KO mice displayed significantly altered propulsion and braking compared to both WT and A53TSyn/NRF2+ mice when all ages were combined (Figure 6B,D,F,H). These data indicate that NRF2 deletion has an effect on the duration of paw surface contact during the propulsion phase of the step motion as well as when the paw returns to the surface after swinging forward during braking. Taken together, these results indicate that NRF2 deletion in combination with the A53TSyn mutation impairs contact time at both the initial and final phases of the stride motion.

## 4. Discussion

This study explored the combined effects of loss of NRF2 and the A53TSyn mutation. We found that knocking out NRF2 in the A53TSyn mouse model of synucleinopathy resulted in decreased mobility with movement fluidity impairment, increased anxiety-related behavior, and reduced TH expression in the striatum without altering pSyn accumulation. The absence of an effect on pSyn expression was somewhat surprising given previous reports of the effect of modulating NRF2 expression in the context of alpha-synuclein pathology. One study reported that AAV-mediated expression of human alpha-synuclein was increased in NRF2KO mice relative to NRF2+ animals [30], and another study found that NRF2 overexpression in astrocytes was associated with reduced alpha-synuclein aggregation in A53TSyn mice [31]. One possible explanation for the discrepancy in the effect of NRF2 on pSyn accumulation in the present study could be the particulars of the models used. Our model exhibited lifelong transgenic expression of alpha-synuclein, rather than AAV-mediated expression induced later in life [30], in conjunction with a global NRF2KO, rather than a cell type-specific one [31]. The fact that these experimental conditions do not appear to affect pSyn accumulation underscores the need for future work validating the effects of loss of NRF2 in other models of synucleinopathy.

NRF2 is the master antioxidant regulatory protein inducing the expression of downstream antioxidant enzymes, which defend the body against ROS. The dysfunction of the antioxidant response system is known to be an influential process in PD progression, accelerating dopaminergic neuronal loss, impairing cell signaling, and disrupting many other homeostatic processes [8]. Modulating the antioxidant response is therefore a significant area of ongoing PD research; the aim of this present study was to elucidate the contribution of NRF2 to motor function within the A53TSyn model of synucleinopathy. Loss of NRF2 resulted in an impaired motor phenotype in A53TSyn mice, and a decrease in TH expression in the striatal region of the brain.

In this study, NRF2 deletion resulted in impaired mobility in A53TSyn mice, demonstrated by total time spent immobile on the OF test as well as through gait abnormalities detected by DigiGait. While to our knowledge this is the first report of the effect of loss of NRF2 in A53TSyn mice, our finding of impaired motor function is consistent with other studies describing the role of NRF2 deletion on mobility and behavior in other mouse models. NRF2 deletion in an MPTP-induced PD model on a C57BL6 background was shown to exacerbate deficits in total distance traveled on the open field test, whereas NRF2 overexpression provided partial protection in the same study [20]. Another study found that NRF2 deficiency reduced physical function and muscle mass in an age-dependent manner, an effect that was attributed to reduced mitochondrial respiration and ROS production in skeletal muscles [22]. Moreover, in another MPTP-induced PD model on a C57BL6 background, a reduction in OS through NRF2 activation improved motor function, suggesting a direct contribution of the NRF2-regulated antioxidant pathway to motor outcomes [21]. The gait analysis in the present study demonstrated that NRF2 deletion significantly influences the duration of paw surface contact during the propulsion phase of the step motion during forward movement. Similarly, during the braking phase of the step motion, wherein the paw returns to the surface after swinging forward, the duration of paw area in contact with the surface per unit time was also found to be significantly different in the fore paws on both the left and the right sides in A53TSyn/NRF2KO relative to A53TSyn/NRF2+. Taken together, these data reflect reduced gait fluidity because of increased dragging and stiffness in the A53TSyn/NRF2KO animals. Although mitochondrial function and oxidative stress were not assessed in our study, it is possible that this reduction in gait fluidity may be the result of muscle deterioration that may have resulted from increased oxidative stress. It would be interesting in future work to compare changes in mitochondrial function and oxidative stress in the brain and muscle in A53TSyn/NRF2KO mice or to explore the effects of a CNS-specific NRF2KO on gait.

We also observed reduced time in the center of the open field in the A53TSyn/NRF2+ and A53TSyn/NRF2KO mice relative to WT mice, although there was no effect of age. In fact, the A53TSyn/NRF2KO mice had an even greater reduction in time in the center than did the A53TSyn/NRF2+ animals, suggesting an additive effect of the loss of NRF2 and overexpression of the transgene. Reduced time in the center of the open field test is consistent with increased anxiety and has been previously reported in A53T transgenic mice [32]. Additionally, treatments that activate the NRF2 pathway have been shown to ameliorate anxiety-related deficits and improve performance in the open field in aged mice as well as in a rotenone-induced PD model [33,34]. The time in the center data should be interpreted with caution, as overall increases in immobility and reductions in distance traveled can impact this metric. While it is possible that increased time immobile in the A53TSyn/NRF2KO mice contributed to the observed reduction in the center time, it is unlikely that there is a confounding effect of distance traveled, as there was not a significant genotype effect observed in that metric. The evidence collected here lends credence to the hypothesis that NRF2 is involved in modulating behavioral deficits in anxiety-related behavior, but further behavioral testing in this model is needed to corroborate this idea.

It is also possible that the changes in motor function detected in the A53TSyn/NRF2KO mice are a consequence of the loss of TH in the brains of those mice, as TH plays an important role in PD and mobility, catalyzing the rate-limiting step in the biosynthesis of dopamine [35]. Our findings are consistent with other reports of the effects of modulating NRF2 activity on dopamine signaling in PD models. In a Thy1-aSyn genetic model of PD with and without NRF2, mice without NRF2 showed increased TH neuronal loss in the striatum and substantia nigra, despite Thy1-aSyn mice typically not demonstrating loss of TH midbrain DA neurons, indicating that NRF2 loss had induced degeneration of midbrain dopaminergic neurons [36]. Additionally, loss of dopamine neurons was exacerbated when MPTP was administered along with the NRF2 inhibitor ML385 [37], and TH expression was decreased in the striatum in an MPTP model following treatment with an NRF2-activating therapy [38].

One hypothesis for how NRF2 may be affecting TH is through its effects on iron homeostasis. Dysregulation of iron homeostasis has been linked to several neurological diseases where dopamine plays a central role, including PD and restless leg syndrome [39], and studies have shown that increased iron concentrations have been observed in the substantia nigra in patients with PD [40]. Iron is also known to be an essential co-factor for TH, and dopaminergic drugs are correlated with increased cellular iron availability. NRF2 is known to have many roles relating to iron, including regulating cell sensitivity to ferroptosis, upregulating the ferritin gene to increase iron efflux, and modulating ferroportin; NRF2KO mice have been reported to have defects in iron utilization [41]. Removal of NRF2 could therefore significantly disrupt the regulation of iron and processes which require iron as a co-factor such as dopamine synthesis. It is likewise possible that NRF2 alters motor function via effects on the muscle. It has been shown that increased oxidative stress can damage muscle tissue, which could contribute to impaired motor function [42]. Although these hypotheses were not evaluated in the present study, investigation in future studies could clarify this question by evaluating the effect of a CNS-specific NRF2KO in A53TSyn mice.

## 5. Limitations of Current Study

There are several limitations to the present study which should be acknowledged. Although we observed pathological and motor differences in this model of synucleinopathy, future studies should seek to confirm these results in other rodent models of PD to substantiate the conclusions drawn from this work. In addition, this study did not include an NRF2KO-only group. Inclusion of this group in future work would lend further credence to the hypothesis that the observed changes are due to the interaction between NRF2 loss and synucleinopathy rather than a global effect of NRF2 deletion. To assess the direct effect of NRF2 removal, future work should also include tests to evaluate relative levels of oxidative stress and downstream alterations to the NRF2 signaling pathway. Finally, increasing group sizes would aid in detecting more subtle differences in motor outcomes, which could have been statistically overlooked in the present study.

## 6. Conclusions

Taken together, this work suggests that NRF2 may play a role in maintaining motor function in the context of synucleinopathy, and therefore interventions that target NRF2 may show promise in improving PD disease outcomes, but further work to elucidate the mechanisms through which these motor effects are elicited is needed.

## Data Availability

The raw data supporting the conclusions of this article will be madeavailable by the authors on request.

## Supplementary Materials

The following supporting information can be downloaded at: https://www.mdpi.com/article/10.3390/antiox15080926/s1, Figure S1: Breeding Schema; Figure S2: Group Distributions; Figure S3: TH expression is reduced in the substantia nigra of A53TSyn/NRF2KO mice. Figure S4: pSyn Aggregation in the Hippocampus and Striatum; Figure S5: DigiGait Paw Placement Metrics Comparison Chart; Figure S6: Propulsion/Breaking Hind Paws.

## Abbreviations

The following abbreviations are used in this manuscript:

PD	Parkinson’s Disease
WT	Wild-Type
TH	Tyrosine Hydroxylase
pSyn	Phosphorylated alpha-synuclein
ROS	Reactive Oxygen Species
NRF2	Nuclear factor erythroid 2-related factor
MO	Months
