# Supplementary material for: NRF2 Deletion Results in Mobility Impairment in A53TSyn Model of Synucleinopathy
Source: Antioxidants (Basel). 2026 Jul 25;15(8):926. doi: 10.3390/antiox15080926 (PMC13509511; doi:10.3390/antiox15080926)
Supplement: Supplementary file 1 [file antioxidants-15-00926-s001.zip › antioxidants-4382720-supplementary.pdf]

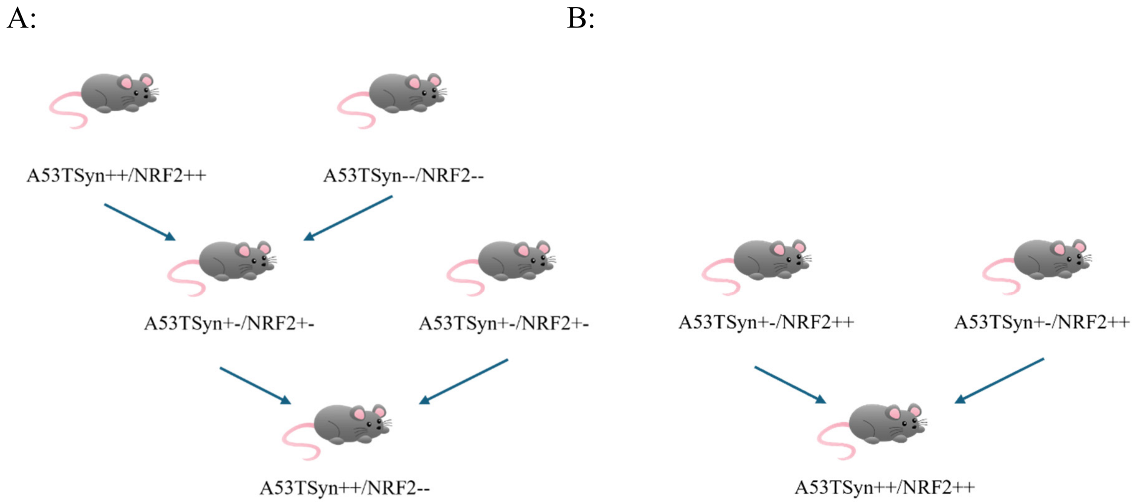

Supplementary Figure S1: Breeding Schema. A: Generation of Homozygous A53TSyn/NRF2KO mice. Homozygous G1 mice for each mutation were paired to generate G2 mice heterozygous for both mutations. G3 mice were homozygous for both mutations (A53TSyn/NRF2KO). B: Generation of homozygous A53TSyn/NRF2+ mice. Heterozygous A53TSyn G1 mice with NRF2 were paired to generate homozygous A53TSyn G2 mice to serve as A53TSyn-NRF2+ controls.

Genotype	4 Months	6 Months	8 Months
WT	8 (4F/4M)	14 (7F/7M)	8 (4F/4M)
A53TSyn/NRF2+	9 (4F/5M)	9 (4F/5M)	8 (4F/4M)
A53TSyn/NRF2KO	14 (7F/7M)	11 (5F/6M)	13 (6F/7M)

Supplementary Figure S2: Animal group distributions.

A

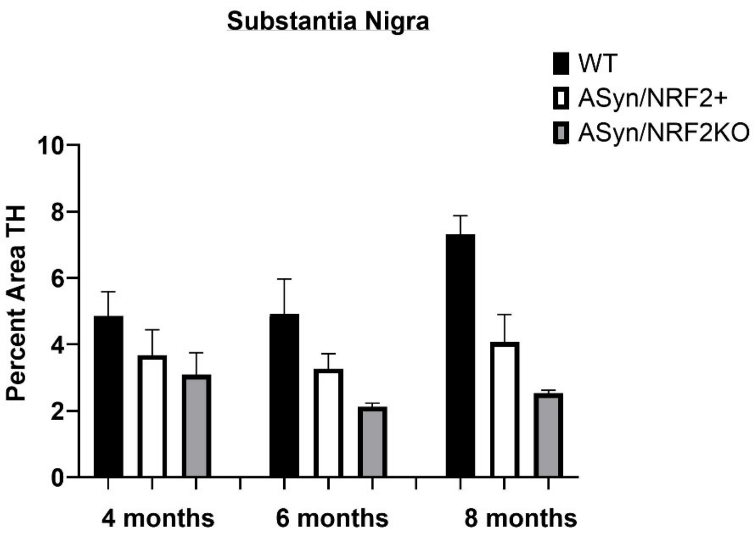

Interaction	F (4, 25) = 1.356	P=0.2774
Genotype	F (2, 25) = 16.68	P<0.0001
Age	F (2, 25) = 2.305	P=0.1206

B

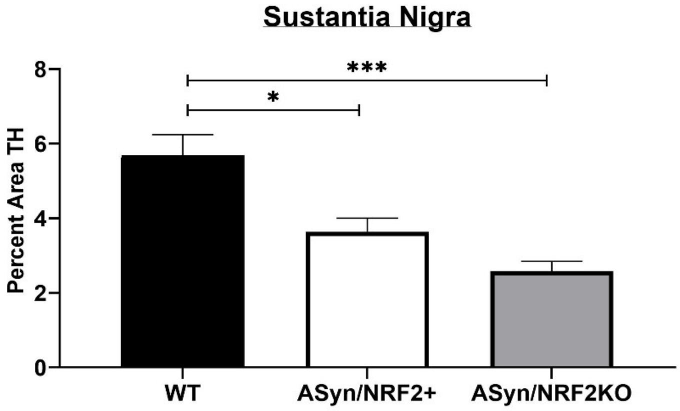

Supplementary Figure S3: TH expression is reduced in the substantia nigra of A53TSyn/NRF2KO mice. TH expression was analyzed in a subset of mice for which the substantia nigra was clearly visible in the slices collected (n=3-4 per condition). **A:** There was a significant effect of genotype on TH expression in the substantia nigra. **B:** Both A53TSyn/NRF2+ and A53TSyn/NRF2KO mice had significantly reduced TH expression in the substantia nigra compared to WT $^{*}p<0.05$, $^{***}p<0.001$

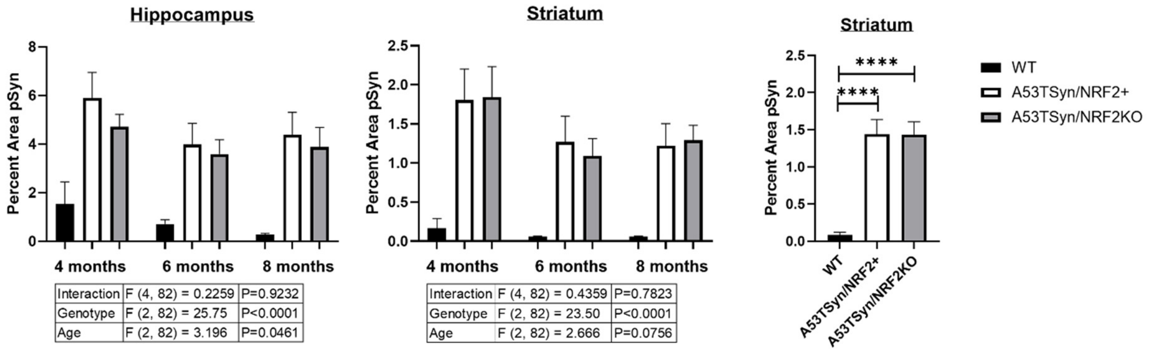

Supplementary Figure S4: Cortical pSyn expression. Hippocampal pSyn aggregation showed a significant interaction between genotype and age, but the interaction was nonsignificant. Striatal pSyn aggregation showed a significant effect in genotype, but not in age nor in interaction. Compiling the average pSyn from all ages showed that there was no statistical difference between A53TSyn/NRF2+ and A53TSyn/NRF2KO, although both were significantly different from WT ($p < 0.0001$).

Digigait Comparison Chart: Paw Placement Metrics

	Left Fore			Right Fore			Left Hind			Right Hind		
**4 Months**												
Overlap Distance												
MIN dA/dT					↓							
MAX dA/dT		↑↑	↑		↑↑	↑						
Percent Shared Stance												
Paw Placement Positioning	↓↓↓↓		↓↓↓				↓↓↓↓		↓↓↓			
Axis Distance							↑↑	↓↓		↓	↑↑	
**6 Months**												
Overlap Distance				↓	↑↑					↓	↑↑	
MIN dA/dT			↓									
MAX dA/dT						↑					↑	
Percent Shared Stance				↓						↓		
Paw Placement Positioning	↓	↑		↓			↓	↑		↓		
Axis Distance							↑	↓↓↓		↓↓	↑↑↑↑	
**8 Months**												
Overlap Distance	↓↓						↓↓					
MIN dA/dT											↓	
MAX dA/dT		↑										
Percent Shared Stance							↑↑↑↑		↑↑↑↑	↑↑↑↑		↑↑↑↑
Paw Placement Positioning	↓↓						↓↓					
Axis Distance							↑↑↑	↓		↓↓		

Legend	
	A53TSyn/NRF2+ vs WT
	A53TSyn/NRF2KO vs A53TSyn/NRF2+
	A53TSyn/NRF2KO vs WT
↑	significantly greater
↓	significantly less than
p < .05	↑
p < .01	↑↑
p < .001	↑↑↑
p < .0001	↑↑↑↑

Supplementary Figure S5: Summary of Significant DigiGait Metrics. Summary of DigiGait metrics which relate specifically to placement of paws on the treadmill. First column gives Overlap Distance, MIN dA/dT, MAX dA/dT, Percent Shared Stance, Paw Placement Positioning, and Axis Distance. 4-month animals are given at the top, 6-month in the middle, and 8-month at the bottom. Each age group is further divided into Left Fore, Right Fore, Left Hind, and Right Hind paws. Significant effects are denoted by color; green gives A53TSyn/NRF2+ relative to wild-type, blue gives A53TSyn/NRF2KO relative to A53TSyn/NRF2+, and red gives A53TSyn/NRF2KO relative to wild-type. Up arrows indicate an effect is significantly greater, while a down arrow indicates the effect is significantly less than. Number of arrows gives p value; one arrow denotes P < .05, two denotes P < .01, three denotes P < .001, and four denote P < .0001.

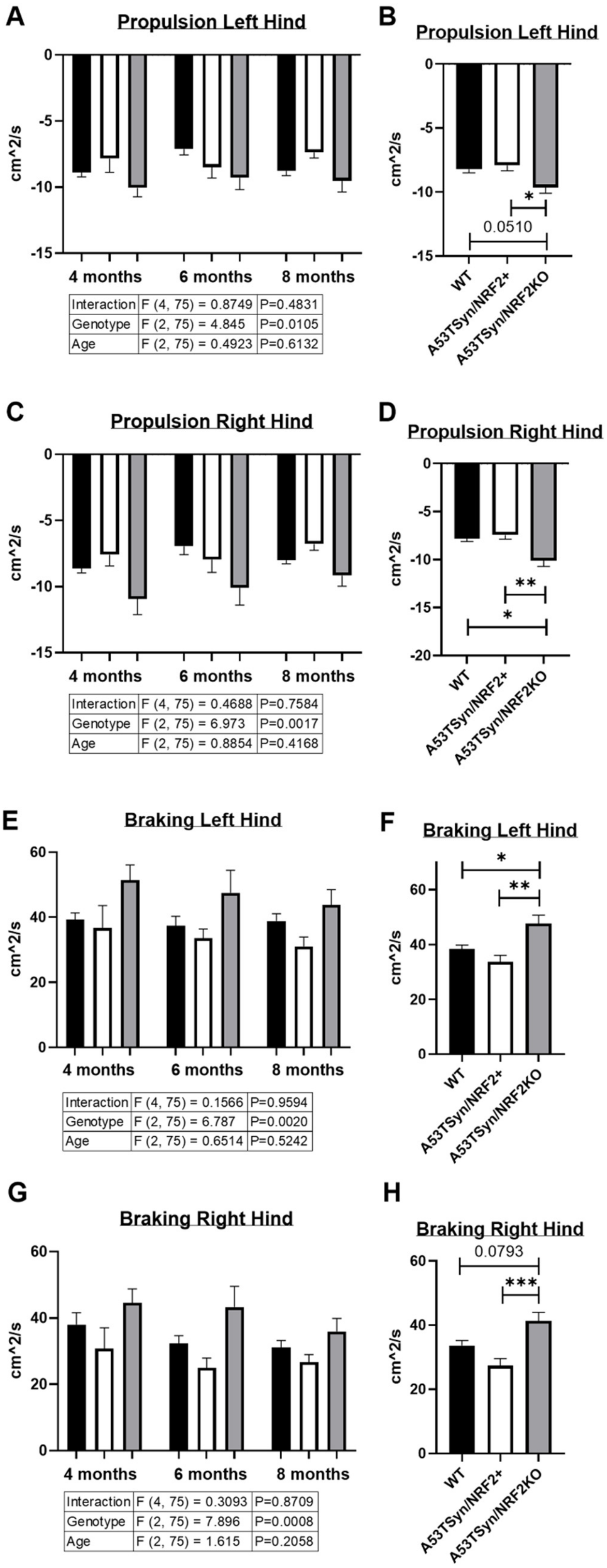

Interaction	F (4, 75) = 0.8749	P=0.4831
Genotype	F (2, 75) = 4.845	P=0.0105
Age	F (2, 75) = 0.4923	P=0.6132

Interaction	F (4, 75) = 0.4688	P=0.7584
Genotype	F (2, 75) = 6.973	P=0.0017
Age	F (2, 75) = 0.8854	P=0.4168

Interaction	F (4, 75) = 0.1566	P=0.9594
Genotype	F (2, 75) = 6.787	P=0.0020
Age	F (2, 75) = 0.6514	P=0.5242

Interaction	F (4, 75) = 0.3093	P=0.8709
Genotype	F (2, 75) = 7.896	P=0.0008
Age	F (2, 75) = 1.615	P=0.2058

Supplementary Figure S6: Propulsion/Breaking Hind Paws. A: Propulsions values during propulsion for the left hind paw by age and genotype. Genotype was a significant effect but age and interaction were not. **B:** Aggregated propulsion values for left hind paw by genotype from all ages. A53TSyn/NRF2KO was not significantly different from WT, although it approached significance ($p = 0.0510$) and was significantly different from A53TSyn/NRF2+ ($p = 0.0126$). **C:** Propulsion values for the right hind paw by age and genotype. Genotype was the only comparison for which there was a significant effect detected. **D:** Aggregated propulsion values for the right hind by genotype from all ages. A53TSyn/NRF2KO was significantly different from WT ($p = 0.0028$) and A53TSyn/NRF2+ ($p = 0.0123$). **E:** Braking values for the left hind paw by age and genotype. Genotype was the only comparison for which there was a significant effect detected. **F:** Aggregated braking values for left hind by genotype from all ages. A53TSyn/NRF2KO was significantly different from WT ($p = 0.0469$) and A53TSyn/NRF2+ ($p = 0.0012$). **G:** Braking values for the right hind paw by age and genotype. Genotype was the only comparison for which there was a significant effect detected. **H:** Aggregated braking values for right hind by genotype from all ages. A53TSyn/NRF2KO approached significance from WT ($p = 0.0793$) and was significantly different from A53TSyn/NRF2+ ($p = 0.0004$). Error bars reflect SEM. *$p<0.05$, **$p<0.01$, ***$p<0.001$ (ANOVA). 4MO WT $n = 7$; 4MO A53TSyn/NRF2+ $n = 7$; 4MO A53TSyn/NRF2KO $n = 14$; 6MO WT $n = 8$; 6MO A53TSyn/NRF2+ $n = 8$; 6MO A53TSyn/NRF2KO $n = 11$; 8MO WT $n = 8$; 8MO A53TSyn/NRF2+ $n = 8$; 8MO A53TSyn/NRF2KO $n = 13$
